# Morphometric traits in the fine-leaved fescues depend on ploidy level: the case of* Festuca amethystina* L.

**DOI:** 10.7717/peerj.5576

**Published:** 2018-09-24

**Authors:** Agnieszka Rewicz, Przemysław Piotr Tomczyk, Marcin Kiedrzyński, Katarzyna Maria Zielińska, Iwona Jędrzejczyk, Monika Rewers, Edyta Kiedrzyńska, Tomasz Rewicz

**Affiliations:** 1 Department of Geobotany and Plant Ecology, Faculty of Biology and Environmental Protection, University of Lodz, Lodz, Poland; 2 Laboratory of Molecular Biology and Cytometry, Department of Agricultural Biotechnology, UTP University of Science and Technology, Bydgoszcz, Poland; 3 Department of Applied Ecology, Faculty of Biology and Environmental Protection, University of Lodz, Lodz, Poland; 4 European Regional Centre for Ecohydrology of the Polish Academy of Sciences, Poland; 5 Department of Invertebrate Zoology and Hydrobiology, Faculty of Biology and Environmental Protection, University of Lodz, Lodz, Poland

**Keywords:** DNA content, Cross-sections of leaves, Morphometric analysis, *Festuca*, Flow cytometry, Genom size

## Abstract

**Background:**

Polyploid specimens are usually characterized by greater exuberance: they reach larger sizes and/or have a larger number of some organs. *Festuca amethystina* L. belongs to the section* Aulaxyper*. Based on morphological features, four subspecies of *F. amethystina* have been already identified. On the other hand, it has two cytotypes: diploid and tetraploid. The main aim of our study was to distinguish morphological differences between the cytotypes of *F. amethystina*, assuming that its phenotype differs significantly.

**Methods:**

The nuclear DNA content was measured by flow cytometry in dry leaves from specimens originating from 13 populations of *F. amethystina*. Several macrometric and micrometric traits of stems, spikelets and leaf blades were taken into account in the comparative analysis of two cytotypes.

**Results:**

In the case of cytotypes, specimens of tetraploids were larger than diploids. The conducted morphometric analysis of leaf cross-sections showed significant differences between the cytotypes.

**Discussion:**

The research has confirmed for the first time that in the case of *F. amethystina* the principle of greater exuberance of polyploids is true. Differences between the cytotypes are statistically significant, however, they are not enough to make easy the distinction of cytotypes on the basis of the measurements themselves. Our findings favor the rule known in *Festuca* taxonomy as a whole, i.e. that the ploidy level can be one of the main classification criteria.

## Introduction

Polyploidization is one of the major methods of plant evolution (Te Beest et al., 2012; Adams & Wendel, 2005; Otto & Whitton, 2000; Ramsey & Schemske, 1998; Bretagnolle & Lumaret, 1995), especially in the case of grasses (Levy & Feldman, 2002; Bretagnolle & Lumaret, 1995). However, it should be emphasized that, after the polyploidization episode, the polyploids undergo selection mechanisms and genome rearrangements (Levy & Feldman, 2002; Bretagnolle & Lumaret, 1995; Macdonald & Chinnappa, 1988; Allendorf, 1979). Polyploidization may be a source of ecological diversification of plant lineages. This can happen when the creation of genetic diversity leads to diversification of ecological niches occupied by plants representing different levels of ploidy (Petit & Thompson, 1999).

Polyploid plants can differ in growth parameters compared to their diploid relatives. There is a correlation between the degree of ploidy and characteristic features of plants, e.g., the size of cells (e.g., the stomata size), the size and features of plant organs—both vegetative (the leaf size, the plant height) and generative (the higher number of inflorescences per plant and flowers per inflorescence) (De Oliveira et al., 2004; Pegtel, 1999; Levin, 2002; Knight & Beaulieu, 2008; Balao, Herrera & Talavera, 2011; Robertson et al., 2010). In general, it is well known that polyploid specimens are usually characterized by greater exuberance (Robertson et al., 2010; Mizukami, 2001; Pegtel, 1999). However, there are exceptions to this rule: the relationship between the ploidy level and the size of organs is not straightforward—it does not have always to be direct or allometric (Otto & Whitton, 2000; Balao, Herrera & Talavera, 2011). Unexpected phenotypic responses differing from the general rule can be a result of developmental trade-offs (Maynard Smith et al., 1985; Prusinkiewicz et al., 2007), changes in the pattern of gene expression (Broz et al., 2009), or even epigenetic effects (Madlung & Wendel, 2013; Madlung et al., 2002; Wang et al., 2004; Balao, Herrera & Talavera, 2011).

Despite these exceptions, numerous attempts were made to determine cytotypes based on morphological features. Macroscopic features were taken into account (e.g., the number of seeds per stem blade, the leaf size, the plant height) as well as microscopic ones (e.g., the size of stomata, the pollen grain sculpture) (Powell, Powell & Tomb, 1977; Bretagnolle & Lumaret, 1995; De Oliveira et al., 2004).

The attempts undertaken had different effects. Most often the morphological distinction of cytotypes turned out to be difficult (e.g., Španiel et al., 2008; Mandáková & Münzbergová, 2008; Ricca M. Beecher et al., 2008; Cires et al., 2009; Lumaret et al., 1987; Amirouche & Misset, 2007; Regele et al., 2017; Macdonald & Chinnappa, 1988). In some cases, important differences between cytotypes were confirmed, but the distinction of ploidy level of individuals with the usage of the found traits remained debatable (e.g., Eliašova, 2008). Differences were often related to microscopic features (difficult for designation in the field), such as the characteristics of stomata or the size and sculpture of pollen grains (e.g., Bretagnolle & Lumaret, 1995; Powell, Powell & Tomb, 1977). A clear distinction of cytotypes is feasible, for example, in the case of *Gallium palustre*, but this was possible only for the diploids and octoploids, the tetraploids were indistinguishable (Kliphuis, Heringa & Hogeweg, 1986).

The evolution of grasses has been accompanied by frequent and repeated genome size gain and loss (Levy & Feldman, 2002; Šmarda et al., 2008), and as a result about 60% of grass species are now classified as polyploid (Goldblatt, 1980), and almost all diploid species of grasses can be considered as paleopolyploid. Among grasses, polyploidy is not only widespread but is also an ongoing process (Levy & Feldman, 2002).

The genus *Festuca* L. is a group of grasses characterized by considerable diversification and worldwide distribution (Inda et al., 2008; Šmarda et al., 2008). Among this genus, species of different ploidy were detected (2×, 4×, 6×, 8×, 10×and 12×; http://data.kew.org/cvalues/). Moreover, about 70% of species are polyploids. The basic chromosome number in *Festuca* is *x* = 7, however, high chromosome number variations were observed (Kaur et al., 2014). In our research, we dealt with one of the species belonging to the mentioned genus: *Festuca amethystina* L. (amethyst fescue). It is a mountain grass that occurs in the area between the Western Alps, Dinarides and Eastern Carpathians (Meusel, Jäger & Weinert, 1965; Jakubowska-Gabara, 1994; Kiedrzyński et al., 2015; Kiedrzyński et al., 2017). The species includes two cytotypes: diploid (2×) with 14 chromosomes and tetraploid (4×) with 28 chromosomes (Inda et al., 2008; Šmarda, 2008). Based on morphological features, four subspecies of *F. amethystina* have already been identified (Markgraf-Dannenberg, 1980; http://ww2.bgbm.org/EuroPlusMed/query.asp): *F. amethystina* L. ssp. *amethystina*, *F. amethystina* ssp. *kummeri* (Beck) Markgr.-Dann., *F. amethystina* ssp. *orientalis* Krajina and *F. amethystina* ssp. *ritschlii* (Hack.) Markgr.-Dann. According to Markgraf-Dannenberg (1980), the four subspecies of *F.amethystina* occur in different geographic regions. The first three subspecies occur mostly in the European mountains and highlands, in subalpine grasslands and in relict limestone pine forests (Coldea, 1991; Kliment et al., 2005; Oberdorfer, 1992). The fourth one, *F. amethystina* ssp. *ritschlii* mostly grows in Polish lowland and upland areas (Jakubowska-Gabara, 1994; Nobis & Piwowarczyk, 2007). This taxon is claimed to be the only subspecies of *F. amethystina* in Poland (Szafer, 1930; Jakubowska-Gabara, 1994; Mirek & Piekoś-Mirkowa, 2009).

However, the taxonomy of the species is not fully clear. Some of the diagnostic features of the subspecies provided by Markgraf-Dannenberg (1980) are quite subjective and difficult to interpret. For example, the following features are given as distinctive: (1) the shape of the upper glume: lanceolate (ssp. *amethystina* and *ritschlii*), ovate-lanceolate (ssp. *kummeri*) or lanceolate to ovate-lanceolate (ssp. *orientalis*); (2) the panicle: dense (ssp. *amethystina* and *kummeri*) or lax (ssp*. ritschlii* and *orientalis*); (3) leaves: smooth (ssp*. ritschlii* and *orientalis*) or scarbid (ssp. *amethystina* and *kummeri*); (4) the number of extravaginal non-flowering shoots: a few (ssp. *amethystina* and *kummeri*) or numerous (ssp. *ritschlii* and *orientalis*). Our preliminary study showed that these diagnostic features, especially dense or lax panicle as well as smooth or scarbid (harsh) leaves, do not follow the above-presented rules.

According to the author, the subspecies *amethystina* can be found on limestones in the Alps and Central Europe; the ssp. *kummeri* on serpentines in the central part of the Balkan Peninsula and West Bulgaria; the ssp. *ritschlii* in North and Central Europe; the ssp. *orientalis* in the East and West Carpathians and the western part of the Balkan Peninsula (Markgraf-Dannenberg, 1980). However, there are also doubts about the allocation of particular subspecies to these geographical regions. For example, specimens identified as belonging to the ssp. *amethystina* were found within the area of ssp. *orientalis* (Indreica, 2007). Some doubts concern also the locations of subspecies in Southern Germany and the Czech Republic. The subspecies is not referred to in the botanical papers from these regions (e.g., Grulich & Grulichová, 1986; Řepka & Roleček, 2002). It seems that the taxonomy of the species is not fully clear and should be revised.

However, field observations confirmed the intraspecific variation of *F. amethystina*. Since we also found different ploidy levels in the analyzed populations, we hypothesized that the most significant phenotypic differences can occur between two cytotypes of *F. amethystina*. The analysis of differences between the cytotypes should be the first step toward a correct description of intraspecific variation of *F. amethystina*.

The main aim of our study was to distinguish quantitative morphological differences between the cytotypes of *F. amethystina*. Plant traits, which are usually dependent on environmental conditions, were analyzed based on the example of individuals which had grown for at least 2 years in a common garden experiment. After this time, we first checked whether in the case of specimens grown in the common conditions the distinction between subspecies according to the Markgraf-Dannenberg (1980) key is possible. In the further analysis, which was based on cytotype identification, we took into account the distinctive features of morphology—the diversity of which was observed in the garden. Also, an analysis of the anatomy of the leaf blade, commonly used in the identification of this group of grasses, was conducted.

## Material and Methods

**Figure 1:**
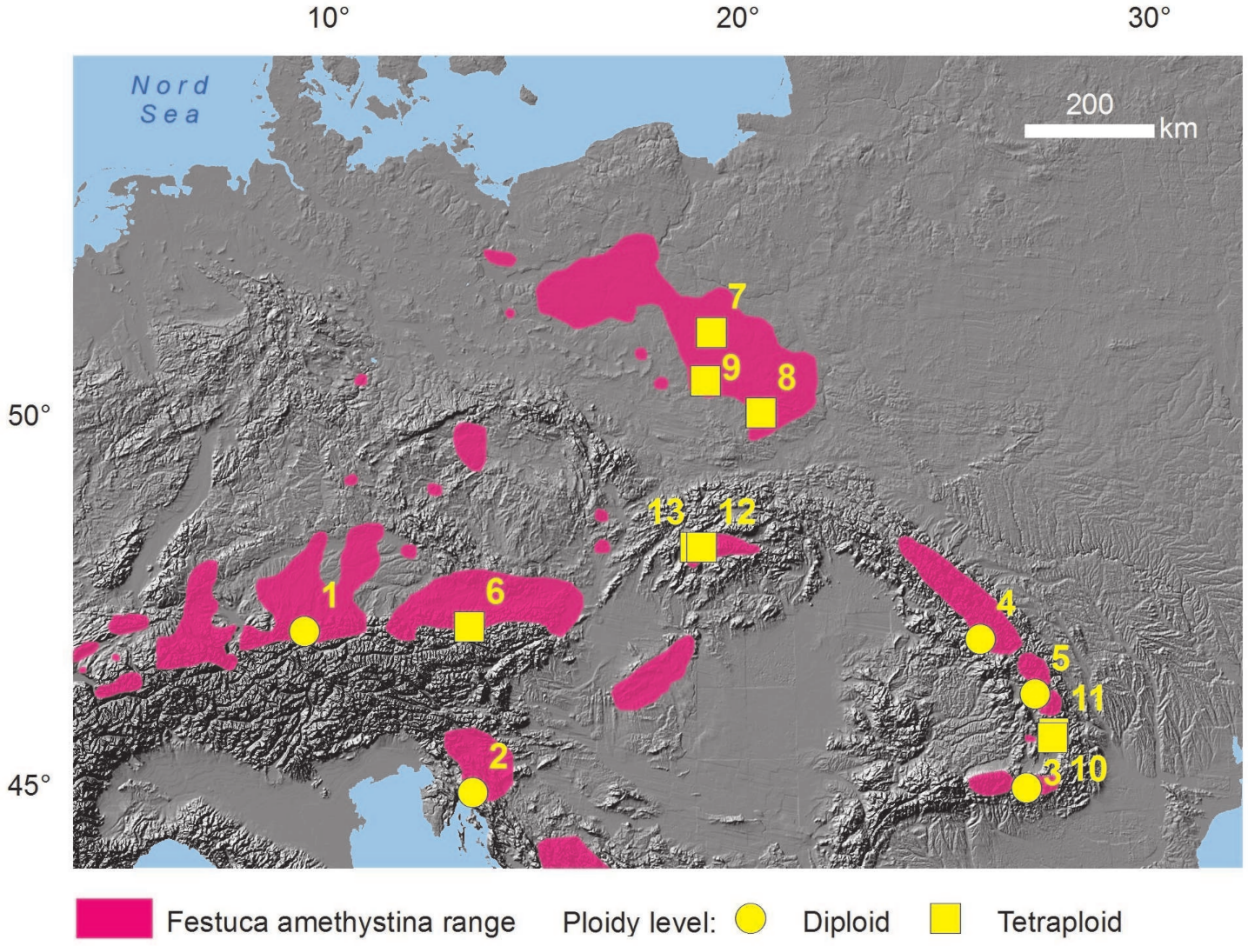
Distribution of the studied populations. Distribution on the basis of *F. amethystina* range (according to Kiedrzyński et al., 2015, changed) and the areas of occurrence of its four subspecies: *ritschlii*, *amethystina, orientalis* and *kummeri* (according to the general description of subspecies in Markgraf-Dannenberg, 1980). Population numbers and ploidy levels according to Table 1.

We analyzed specimens obtained from 13 *F. amethystina* populations (Table 1, Fig. 1). The plant material was collected during field trips to Romania, Croatia and Germany in 2014 and Poland in 2014 and 2015. The collection of dry material was deposited in the Laboratory of Plant Ecology and Adaptation at the Faculty of Biology and Environmental Protection, University of Lodz. Living individuals (fragments of clumps) were also brought from the expeditions, they were planted in the Experimental-Didactic Garden of the Faculty of Biology and Environmental Protection, University of Lodz. A field permit for collecting material was granted by the decisions of the Regional Directors for the Environmental Protection: WPN.6205.170.2014.KLD; WPN-II.6400.14.2014.KW2; WPN.I.6400.21.2014.AC.

### Determination of subspecies

Because the main interspecific variation of *F. amethystina* is based on subspecies determination, we checked this possibility first. The determination of specimens, collected from different populations, to subspecies was made accordingly to the key developed by Markgraf-Dannenberg (1980) (Tables 2 and 3). The characteristic quantitative features of subspecies distinguished by Markgraf-Dannenberg (1980) are presented below:

(1) subsp. **amethystina**:
  (a) 1 Extravaginal non-flowering shoots few, panicle dense; leaves scarbid
  (b) 2 Upper glume 3–4.2 × 0.9–1.1 mm, lanceolate
(2) subsp. **kummeri**:
  (a) 2 Upper glume 3.5–5.4 × 1–1.6 mm, ovate-lanceolate
(3) subsp. ritschlii:
  (a) 1 Extravaginal non-flowering shoots numerous; panicle lax; leaves smooth
  (b) 3 Stems 75–100 cm; panicle 14–17 cm; upper glume lanceolate
(4) subsp. **orientalis**:
  (a) 3 Stems 30–60 cm; panicle 4–15 cm; upper glume lanceolate to ovate-lanceolate

In five cases, we could not assign the analyzed specimens to the combination of features given in the first step of the key (Table 2). The second step of the subspecies determination enabled diagnosis only in 1 case (Table 3). The conclusion can be drawn that the division of the studied specimens of *F. amethystina* into the subspecies on the basis of the used key is doubtful. However, since the subspecies description made by Markgraf-Dannenberg (1980), some crucial localities have been discovered beyond the range known at that time. For example, the subspecies *ritschlii* was known in Poland, almost exclusively, from one region (Kiedrzyński et al., 2017), as well as discovery of the new populations in Romania (Indreica, 2007), which was mentioned above. It is possible that the limited knowledge about the intraspecific variation influenced the choice of diagnostic features by Markgraf-Dannenberg (1980). Hence, in our study the ploidy level as a determinant was selected for the further morphometric analysis.

**Table 1:** List of studied populations of *F. amethystina*. Designation of specimens to subspecies was made on the basis of geographical location according to Markgraf-Dannenberg (1980).

No.	Locality name	Country	Ploidy level inferred by FCM	Mean 2C DNA [pg ± SD]
1	Garmisch-Partenkirchen	Germany	2	7.17 ± 0.162
2	Guślica	Croatia	2	6.99 ± 0.243
3	Caraiman	Romania	2	7.20 ± 0.359
4	Corongis	Romania	2	6.96 ± 0.390
5	Hasmasul Mare	Romania	2	7.24 ± 0.332
6	Mayrwinkl	Austria	4	13.12 ± 0.584
7	Dąbrowa Grotnicka	Poland	4	13.90 ± 0.765
8	Grzywy Korzeckowskie	Poland	4	14.19 ± 1.153
9	Wola Wydrzyna	Poland	4	12.57 ± 0.122
10	Petriceni	Romania	4	13.87 ± 1.196
11	Valea Seaca	Romania	4	13.45 ± 0.636
12	Borisov	Slovakia	4	13.54 ± 0.727
13	Tlsta	Slovakia	4	13.63 ± 0.729

**Table 2:** First step of subspecies diagnosis accordingly to Markgraf-Dannenberg (1980). Small number of extravaginal non-flowering shoots, dense panicle and scarbid leaves indicate subsp. *amethystina* or *kummeri* (*ameth./kum.*), the opposite features indicate subsp. *ritschlii* or *orientalis* (*rits./orient.*); the results of determination of *F. amethystina* specimens from different populations are in the columns labeled Diagnosis: P, positive (the features of specimens fit to one of the key sets of traits); N, negative (the features do not fit).

No.	Locality name	Extravaginal non-flowering shoots		Panicle		Leaves		Diagnosis	
		few	numerous	dense	lax	scabrid	smooth	*ameth./kum.*	*rits./orient.*
1	Garmisch-Partenkirchen		+	+		+		N	N
2	Guślica		+	+			+	N	N
3	Caraiman		+		+		+	N	P
4	Corongis		+		+		+	N	P
5	Hasmasul Mare		+		+		+	N	P
6	Mayrwinkl		+		+		+	N	P
7	Dąbrowa Grotnicka	+		+		+		P	N
8	Grzywy Korzeckowskie	+		+		+		P	N
9	Wola Wydrzyna	+		+		+		P	N
10	Petriceni		+		+	+		N	N
11	Valea Seaca	+		+		+		P	N
12	Borisov	+			+	+		N	N
13	Tlsta	+			+	+		N	N

**Table 3:** The second step of subspecies diagnosis accordingly to Markgraf-Dannenberg (1980). The results of determination of *F. amethystina* specimens from different populations are in the columns labeled Diagnosis: P, positive (the features of specimens fit to one of the key sets of traits); N, negative (the features do not fit); diagnosis were completed only in case of populations for which the first step diagnostic features allowed it (see Table 2).

No.	Locality name	Upper glume (mm)		Upper glume		Diagnosis		Stem (cm)		Panicle (cm)		Upper glume		Diagnosis	
		3–4.2 × 0.9–1.1	3.5–5.4 × 1–1.6	lanceolate	ovate-lanceolate	*amethystina*	*kummeri*	75–100	30–60	14–17	4–15	lanceolate	lanc. to ovate-lanceolate	*ritschlii*	*orientalis*
3	Caraiman	N		+	+	N	N	N			+			N	N
4	Corongis	N		+	+	N	N	N			+			N	N
5	Hasmasul Mare	N			+	N	N	N			+		*+*	N	N
6	Mayrwinkl	P		+		P	N	N			+			N	N
7	Dąbrowa Grotnicka	N				N	N	P			+	*+*	*+*	N	N
8	Grzywy Korzeckowskie	N			+	N	N	P			+		*+*	N	N
9	Wola Wydrzyna	N			+	N	N	N			+	*+*		N	N
11	Valea Seaca	N				N	N	N			+	*+*	*+*	N	N

### Flow cytometry analysis

The nuclear DNA content was measured in dry leaves of all *Festuca* accessions. The samples for a flow cytometric analysis were prepared according to the procedure described by Jędrzejczyk & Śliwinska (2010). The plant material was chopped with a razor blade in a Petri dish in the presence of one ml of nucleus-isolation buffer (200 mMTris; four mM MgCl_2_ ×6H_2_O; 0.5% (v/v) Triton X-100; pH 7.5; (Zenkteler & Jędrzejczyk, 2012) with the addition of propidium iodide (PI 50 µg/ml) and ribonuclease A (RNase A 50 µg/ml). The chopped plant material was filtered through a nylon filter with a mesh diameter of 50 µm and then the cell suspension was analyzed using a CyFlow Ploidy Analyser (Sysmex Partec GmbH, Görlitz, Germany) equipped with a high-grade solid state laser with green light emission at 532 nm, as well as with side (SSC) and forward (FSC) scatters. For each sample, the nuclear DNA content in 5,000–7,000 nuclei was measured with the usage of the linear amplification. Out of each population, 10 individuals were analyzed. The obtained histograms were evaluated by the CyFlow Cube program (Sysmex Partec GmbH, Görlitz, Germany). The nuclear DNA content was calculated by the linear relationship between the ratio of the 2C peak positions of *Festuca* genotypes and the internal standard *Pisum sativum* cv. Set (2C = 9.11 pg; (Śliwińska, Zielińska & Jędrzejczyk, 2005; Rewers & Jędrzejczyk, 2016) on the histogram of fluorescence intensities. The coefficient of variation (CV) of the G_0_/G_1_ peak of *F. amethystina* ranged between 2.29% and 5.91%.

### Analysis of morphometric traits of *F. amethystina*

We measured the chosen organs of 39 diploid and 34 tetraploid individuals (one measurement per individual) of *F. amethystina* from 13 analyzed populations. The measurements were conducted on the plant material from living specimens collected during field expeditions and planted in the Experimental-Didactic Garden of the Faculty of Biology and Environmental Protection. This way all measurements were made on individuals which had grown under the same conditions for 2 or 3 years.

(a) ***Macrometric traits***

Measurements of general plant traits

Macrometric traits were selected based on a two-year observation of specimens growing in the garden. Each individual was described by the following four chosen qualitative traits: the length of the leaf (cm)—LLe, the length of the stalk (cm)—LS, the length of the panicle (cm)—LPa, and the number of spikelets—NS (Table 4). Morphological terminology of the taxon description was adopted according to Ellis (1976).

**Table 4:** Macrometric characteristics of cytotypes of *F. amethystina*.

	Ploidy level	*n*	$\bar {x}$	Med	Min	Max	SD	CV	UMann–Whitney test *p*
**(A)**									
Length of leaf (cm)	D	39	24.61	24.70	15.30	40.50	6.42	26.07	*p* < 0.001
	T	34	37.16	36.00	25.50	58.00	6.91	18.59	
Length of stalk (cm)	D	39	62.25	61.80	36.30	95.00	12.95	20.81	*p* < 0.001
	T	34	85.15	85.50	56.50	119.00	15.55	18.26	
Length of panicle (cm)	D	39	8.64	8.50	3.00	12.50	2.05	23.71	*p* < 0.001
	T	34	11.82	11.50	6.50	19.30	3.08	26.04	
Number of spikelets	D	39	47.44	45.00	21.00	79.00	14.39	30.34	*p* = 0.777
	T	34	46.91	47.00	19.00	80.00	16.00	34.11	
**(B)**									
Lower glume (mm)	D	25	3.71	3.50	2.32	6.70	0.77	20.86	*p* < 0.001
	T	40	4.74	4.89	2.50	6.54	0.85	17.92	
Upper glume (mm)	D	25	3.21	3.33	1.50	6.70	0.93	28.93	*p* < 0.001
	T	40	4.18	4.52	1.50	6.13	1.15	27.50	
Lemma (mm)	D	25	4.05	4.23	2.08	6.32	0.68	16.72	*p* < 0.001
	T	40	4.78	4.74	3.36	6.47	0.52	10.81	
Palea (mm)	D	25	3.59	3.59	1.02	5.35	0.88	24.55	*p* < 0.001
	T	40	4.20	4.33	1.01	6.50	1.05	25.09	

**Notes.**

A measurements of general plant traits B measurements of spikelet elements x arithmetic means med median min minimum max maximum SD standard deviation CV (%) variation coefficients D diploid T tetraploid

Measurements of spikelet elements

We measured the chosen traits of 30 spikelets from each population. Each spikelet was described by four qualitative traits: the length of lower glumes (mm)—LLG, the length of upper glumes (mm)—LUG, the length of the lemma (mm)—LL, and the length of the palea (mm)—LP. The biometric traits analysis was performed using a stereoscopic microscope Nikon SMZ-800 DS-Fi with a camera and Cool View software (semi-automatic biometric traits measurements).

(a) ***Analysis of leaf cross-sections***

**Figure 2:**
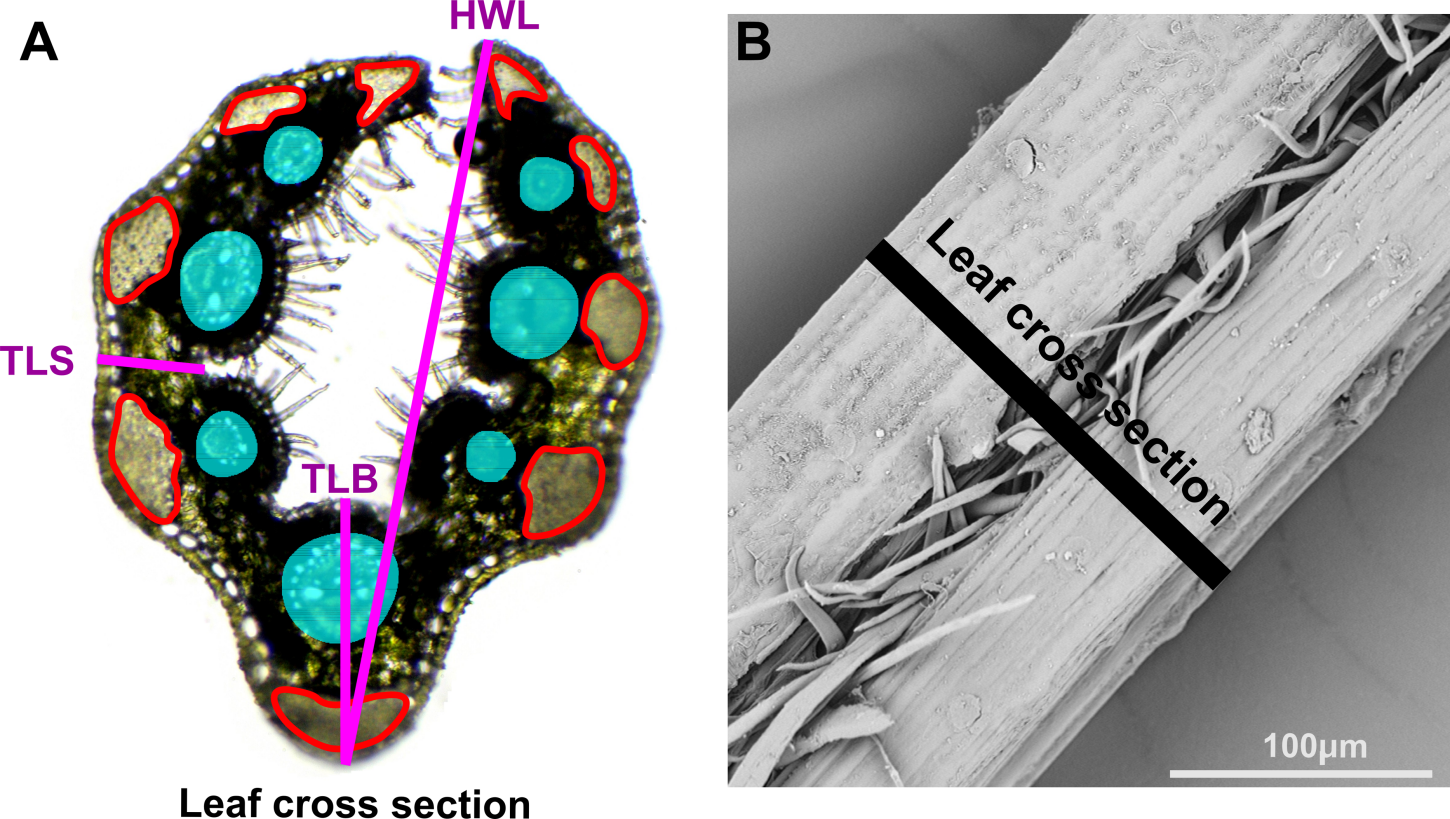
Measurements of the leaf cross-section of *F. amethystina*, (A); (B) traits selected for biometric analysis. HWL, the half of the width of the leaf; TLB, the thickness of the leaf on the bend; TLS, the thickness of the leaf on the side; SCSB, the sum of the circumferences of sclerenchyma bands, marked red; SFVB, the sum of the field of circles in which the vascular bundles could be inscribed—marked blue. Photos by M Kiedrzyński and A Rewicz.

In the genus *Festuca*, the analysis of the leaf cross-section is a widely accepted method of species distinction, hence we checked if it was important also in the determination of cytotypes in *F. amethystina*. In order to determine the proper traits, we analyzed thematic papers (e.g., Pawlus, 1983–1985; Wilson, Thompson & Hodgson, 1999; Bednarska, 2012;  Martínez-Sagarra, Abad & Devesa, 2017). Five traits selected for the biometric analysis were as follows: HWL—the half of the width of the leaf; TLB—the thickness of the leaf on the bend; TLS—the thickness of the leaf on the side; SCSB—the sum of the circumferences of sclerenchyma bands; and SFVB—the sum of the field of circles in which the vascular bundles could be inscribed (Fig. 2). The leaves chosen to the cross-sections were taken from three randomly-selected tillers from the cluster (i.e., one specimen); these were mature leaves that grow from the basal parts of non-flowering tillers. The cross-sections were made by hand with the usage of surgical blades, the cuts were made in the middle third of blades. The analysis of the cross-sections was conducted under a light microscope. After the cross-section, the preparation was analyzed and documented as a digital picture in ToupView software.

### Statistical analyses

The following statistics were analyzed: arithmetic mean (x), median (med), maximum and minimum values (max and min), standard deviation (SD), and coefficient of variation (CV). Conformity of the data distribution with the normal distribution was verified using the Shapiro–Wilk and Kolmogorov–Smirnov tests. Differences in morphometric traits were analyzed with the non-parametric UMann–Whitney test. The significance level for those statistical analyses was *P* < 0.05. To detect patterns in differences in morphometry of cytotypes of *F. amethystina*, the Principal Components Analysis was used (PCA; Van Emden, 2008). The analysis was performed on a plot basis with the usage of the morphological traits. Calculations were made with the software packages STATISTICA PL. ver. 14 ( Stat-Soft Inc., 2011) (Van Emden, 2008).

## Results

### Flow cytometry analysis

The flow cytometric measurements indicated two different inferred ploidy levels within *Festuca amethystina* accessions, corresponding to different values of the DNA amount. Both diploid and tetraploid plants were observed. In diploid plants, the genome size (2C) ranged from 6.96 pg to 7.24 pg. The mean nuclear DNA content (2C) for four diploid plants that represented the populations from Romania and Croatia was 7.10 pg. For tetraploid plants, the 2C nuclear DNA content ranged from 12.57 pg to 14.19 pg and those minimum and maximum values were obtained for the populations from Poland (Table 1).

### General macrometric traits of cytotypes

**Figure 3:**
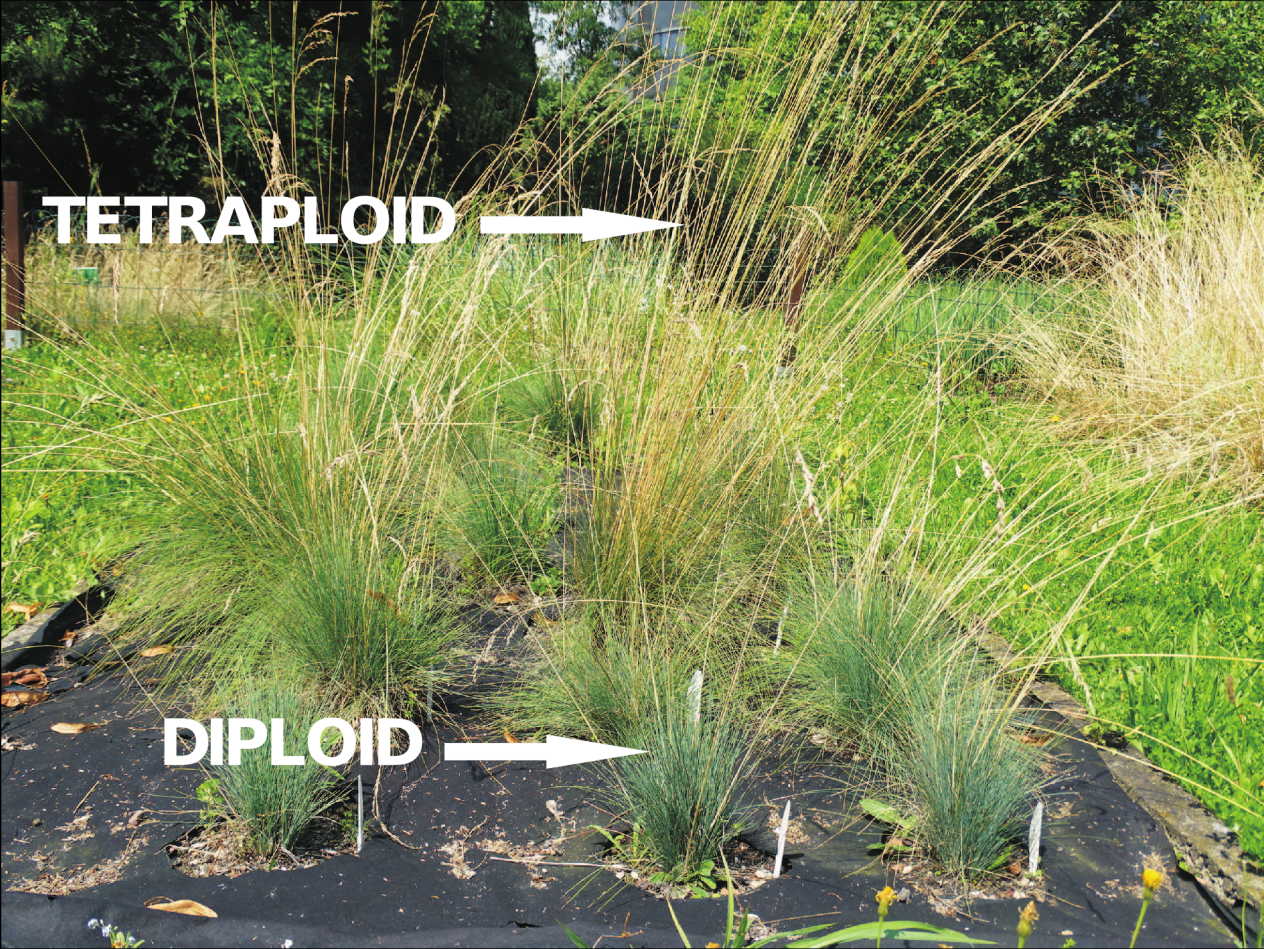
Specimens of *F. amethystina* in the Experimental Garden of the Faculty of Biology and Environmental Protection, University of Lodz. Photo M. Kiedrzyński 2015.

Tetraploids were larger than diploids. The length of the stalk varied from 36.3 to 95.0, and from 56.5 to 119.0 cm for diploids and tetraploids respectively (Tables 4 and 5, Fig. 3). A similar pattern was observed in the length of the leaf, which varied from 15.3 to 40.5 cm, 25.5 to 58.0 cm for diploids and tetraploids respectively. Moreover, higher average values of the examined features of tetraploids were confirmed (except the mean number of spikelets, where diploid and tetraploid plants have similar results: about 47). The highest coefficient of variation was observed for the number of spikelets, it ranged from 30.34 to 34.11%. The lowest coefficient of variation in all the analyzed features was observed for the lemma (ranged from CV = 10.81% in tetraploids to CV = 16.72% in diploids).

**Table 5:** Range of biometric features of *F. amethystina* cytotypes: (A) general plant traits, (B) spikelet elements and (C) leaf cross-sections.

	Ploidy level	Min	Max
**(A) Measurements of general planttraits**			
Length of leaf (cm)	D	15.30	40.50
	T	25.50	58.00
Length of stalk (cm)	D	36.30	95.00
	T	56.50	119.00
Length of panicle (cm)	D	3.00	12.50
	T	6.50	19.30
Number of spikelets	D	21.00	79.00
	T	19.00	80.00
**(B) Measurements of spikelet elements**			
Lower glume (mm)	D	2.32	6.70
	T	2.50	6.54
Upper glume (mm)	D	1.50	6.70
	T	1.50	6.13
Lemma (mm)	D	2.08	6.32
	T	3.36	6.47
Palea (mm)	D	1.02	5.35
	T	1.01	6.50
**(C) Morphometric characteristics of leaf cross-sections**			
The half the width of the leaf (HWL) (mm)	D	0.44	0.83
	T	0.58	0.91
The thickness of the leaf on the bend (TLB) (mm)	D	0.18	0.31
	T	0.22	0.35
The thickness of the leaf on the side (TLS) (mm)	D	0.12	0.22
	T	0.12	0.23
The sum of the circumferences of the sclerenchyma bands (SCSB) (mm)	D	1.07	2.42
	T	1.40	3.13
The sum of the circle field of vascular bundles (SFVB) (mm)	D	18.03	67.27
	T	22.36	72.72

**Figure 4:**
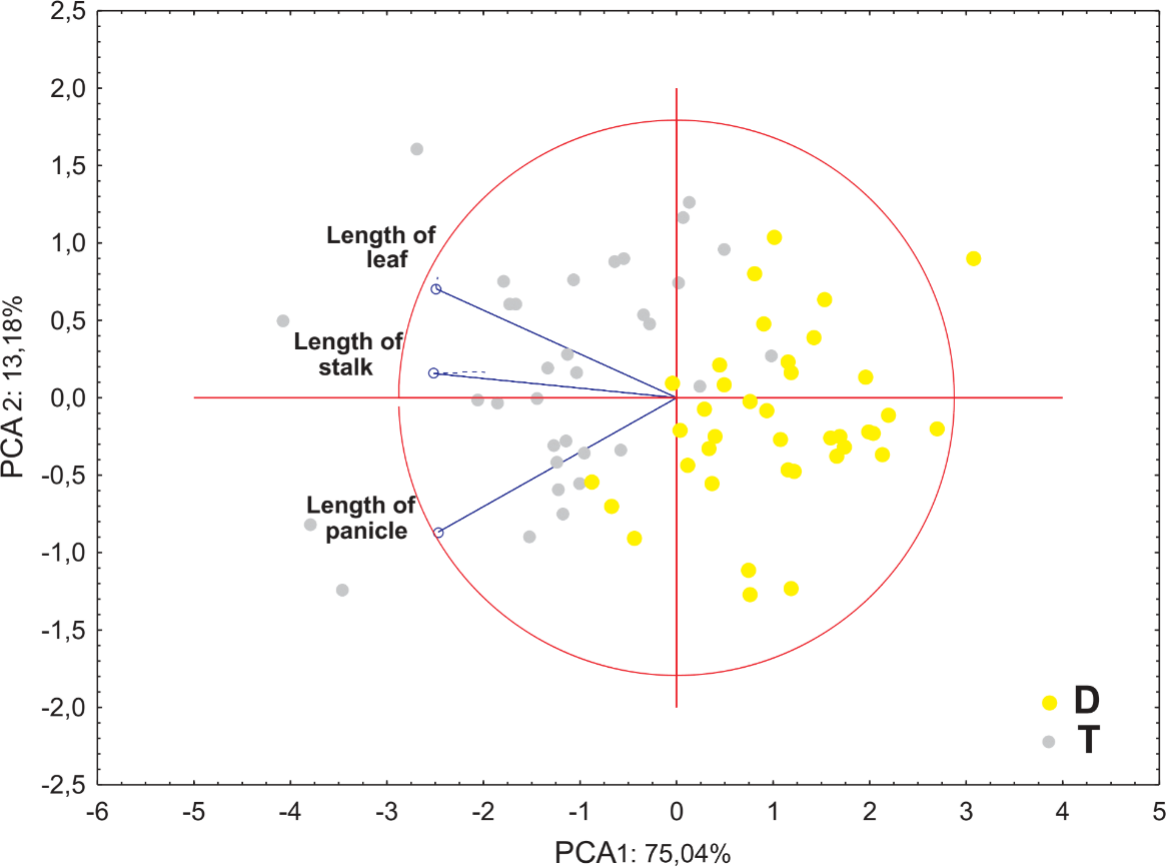
Ordination diagrams of the Principal Component Analysis (PCA) of cytotypes of *F. amethystina* based on the length of the leaf, stalk and panicle.

**Figure 5:**
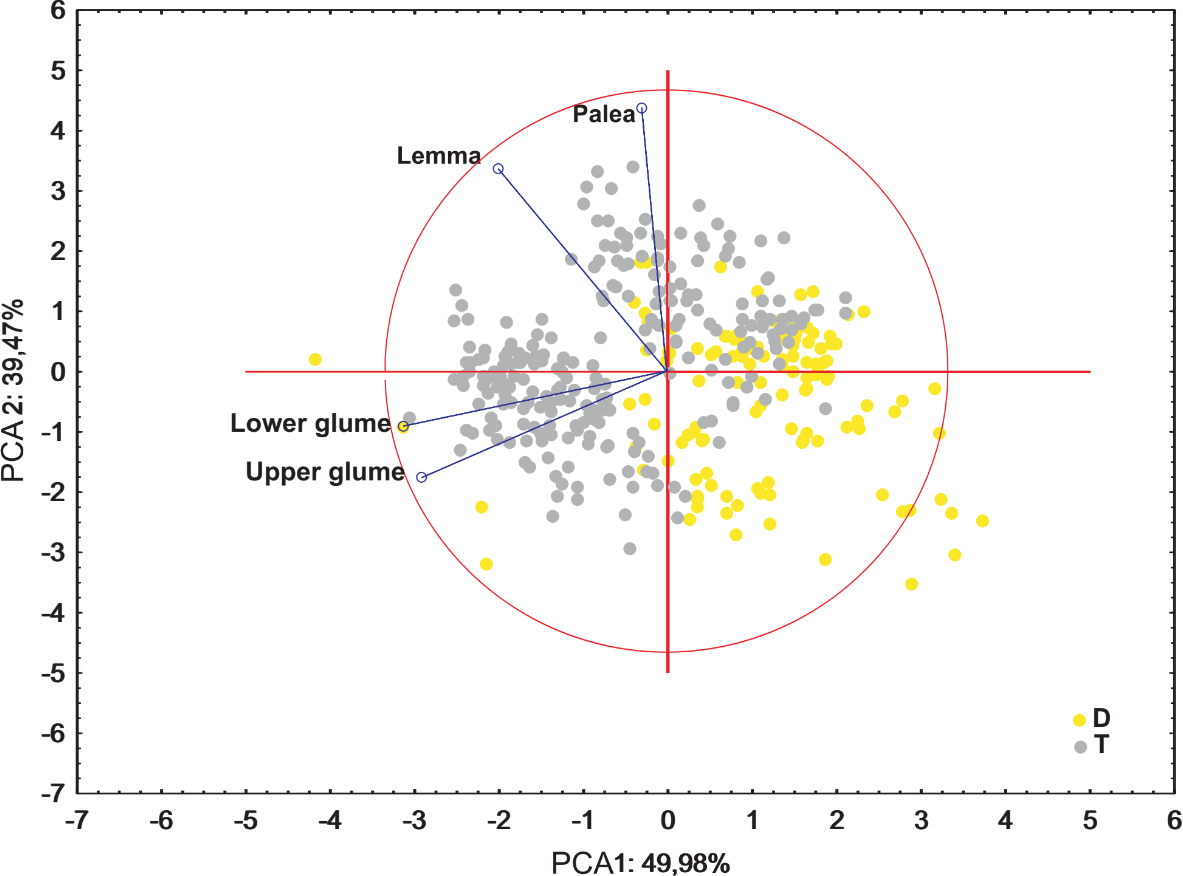
Ordination diagrams of the PCA of *F. amethystina* cytotypes based on the lower glume, the upper glume, the lemma and the palea.

Ordination diagrams of Principal Component Analysis (PCA) of cytotypes based on the measurements of the lower glume, the upper glume, the lemma and the palea revealed that the first two principal components explained 89.45% of the total variance. The second PCA ordination based on the length of the leaf, stalk and panicle explained 88.22% of the variance. Both diagrams showed that diploid and tetraploid specimens were located on the opposite sides of continuous variability of the examined features (Figs. 4 and 5).

**Table 6:** Morphometric characteristics of leaf cross-sections in cytotypes of *F. amethystina*.

Feature	Ploidy level	$\bar {X}$	Med	Min	Max	SD	CV	UMann–Whitney test *p*
The half the width of the leaf (HWL) (mm)	D	0.61	0.60	0.44	0.83	0.08	13	*p* < 0.0002
	T	0.69	0.68	0.58	0.91	0.08	12	
The thickness of the leaf on the bend (TLB) (mm)	D	0.24	0.24	0.18	0.31	0.03	12	*p* < 0.0004
	T	0.27	0.26	0.22	0.35	0.03	11	
The thickness of the leaf on the side (TLS) (mm)	D	0.16	0.16	0.12	0.22	0.03	16	*p* < 0.0009
	T	0.19	0.19	0.12	0.23	0.02	12	
The sum of the circumferences of the sclerenchyma bands (SCSB) (mm)	D	1.73	1.73	1.07	2.42	0.37	22	*p* < 0.0005
	T	2.18	2.07	1.40	3.13	0.46	21	
The sum of the circle field of vascular bundles (SFVB) (mm)	D	36.00	34.14	18.03	67.27	11.90	33	*p* < 0.0001
	T	48.16	45.54	22.36	72.72	11.01	23	

**Notes.**

x arithmetic means med median min minimum max maximum SD standard deviation CV (%) variation coefficient D diploid T tetraploid

### Morphometric analysis of leaf cross-sections in cytotypes

All elements of leaf cross-sections of tetraploids were larger than diploids. The length of the half of the width of the leaf varied from 0.44 to 0.83 and from 0.58 to 0.91 for diploid and tetraploid plants respectively (Tables 5 and 6).

The highest coefficient of variation was observed for the sum of the field of vascular bundles, it ranged from 33% (for diploids) to 23% (for tetraploids), and for the sum of the circumferences of sclerenchyma bands (22%, 21% diploids, tetraploids respectively). The lowest coefficient of variation was observed for the thickness of the leaf on the band (ranged from CV = 11% - tetraploids to CV = 12% - diploids) and the half of the width of the leaf (ranged from CV = 12% - tetraploids to CV = 13%—diploids).

**Figure 6:**
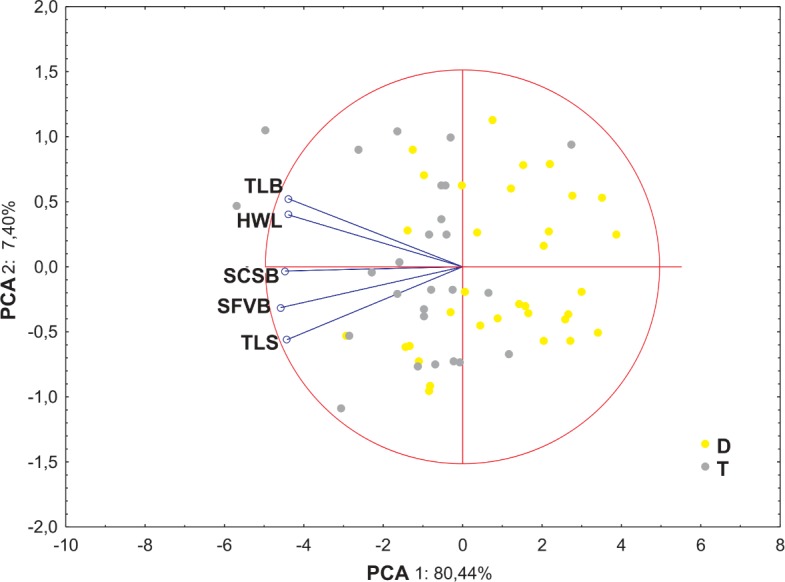
Ordination diagrams of Principal Component Analysis (PCA) of cytotypes of *F. amethystina* based on five morphometric traits of leaf cross-sections. Abbreviations of morphometric traits according to Table 6.

The PCA analysis based on five morphometric traits of leaf cross-sections revealed that the first two component accounted for approx. 88% of the total variance. The ordination shows that distinction between the cytotypes is not so clear as in the previous analysis, however, the main pattern is similar—diploid and tetraploid plants are located rather on the opposite sides of the ordination space (Fig. 6).

## Discussion

It is well proven that in many cases polyploid plants are characterized by a large size and great vigor of cells, leaves, flowers, and fruit compared to their diploid relatives (Tamayo-Ordóñez et al., 2016). They usually have more opportunities to respond to changing environmental conditions and have more chance for expansion to newly formed habitats. This is probably related to an increased degree of heterozygosity, which can be an essential factor for growth, development and adaptability of polyploids (Tamayo-Ordóñez et al., 2016).

In the case of the section *Aulaxyper*, which includes *F. amethystina*, the species used to be classified into species groups according to important morphological characters, which, however, vary among authors. Moreover, even the study based on molecular data does not provide a reliable and detailed synthetic view on their evolution (Šmarda et al., 2008).

As we have indicated in the case of *F. amethystina*, intraspecific variation based on morphological traits should be updated. We have shown that the previous key is not working. However, the clear differences between plants are visible in the common garden cultivation. After the study of this variability in terms of the degree of ploidy, it turned out to be statistically significant.

We have confirmed for the first time the principle of greater exuberance of polyploids in the case of studied species. In the macrometric traits, statistically significant differences between the diploids and tetraploids in the size of vegetative and generative organs are visible. Tetraploid plants have longer leaves, stalks and panicles. The parts of spikelets such as the palea, the lemma, the lower glume and the upper glume are also larger.

We also analyzed the anatomy of leaf cross-section. Also, in this case, the differences between the cytotypes turned out to be statistically significant. The analysis indicates larger thickness of leaves in tetraploid plants. The anatomical structure of leaves, such as sclerenchyma bands and vascular bundles, is also larger in tetraploids. Thus inferred ploidy level is significantly related to the morphological and anatomical features of studied grass. However, it should be noted that the features found correspond to the overall size of the plant, the larger plants have larger, e.g., sclerenchyma, bands and do not have a significantly different internal structure. Moreover, we have shown that it is necessary to conduct an analysis of the intraspecific variation based on the degree of ploidy.

As described above, the carried out research indicates that in the case of *F. amethystina* the ploidy level is one of the basic classification and description criteria. This situation seems to be true also for other members of the genus *Festuca*. For example, Easton (1977) found that in the case of isogenic diploid and tetraploid forms of meadow fescue *Festuca pratensis*, the tetraploids had larger leaves and tillers but a lower relative growth rate. For most vegetative characters studied, the tetraploid phenotypic variance was greater than diploid and was attributed to multi-allelic interactions created by the tetraploid state (Easton, 1977). Šmarda & Stančik (2006) even claim that without knowledge of the inferred ploidy level, almost no systematic and taxonomic study can be done on this genus of grasses.

To summarize, we have demonstrated the need to start a taxonomic revision of the species from the analysis of variability resulting from polyploidization. Other researchers have indicated similar results, and it may turn out that this variation is more appropriate for the first step of analysis. Our findings support general statements on the section *Aulaxyper* in the genus *Festuca*. In the current species concept and in *Festuca* taxonomy as a whole, the ploidy level plays a very important role and is often the main classification criterion. This is especially manifested in larger and taxonomically problematic groups such as the *Festuca ovina* and *Festuca pallens*. In some cases, no further than the ploidy level division was possible, and the morphological and geographical types distinguished in earlier studies were merged within each ploidy level (Šmarda & Stančik, 2006). In the case of all tetraploid plants of *F. pallens*, for practical purposes and due to only minor morphological differences, all tetraploid populations are included in one polymorphic species (Šmarda & Stančik, 2006).

## Conclusions

In the presented research, the importance of ploidy level for both the morphological and anatomical structure of *F. amethystina* has been demonstrated for the first time. Our study clearly shows that existing criteria for distinguishing subspecies could be misleading and the morphological assignment of plants is much easier according to the ploidy level. The discussed issue requires further, more in-depth research, also with the use of methods other than biometrics. We will seek to continue the research, taking into account habitat niches and genetic differences (phylogenetic) in the whole species range in order to comprehensively explain relationships within and between the cytotypes of *F. amethystina*.

##  Supplemental Information

10.7717/peerj.5576/supp-1 Table S1 The values of measurement of spikelet elements of F. amethystina We measured the chosen traits of 30 spikelets from each population. Each spikelet was described by four traits: the length of lower glumes (mm) –LLG, the length of upper glumes (mm) –LUG, the length of the lemma (mm) –LL, the length of the palea (mm) –LP.

10.7717/peerj.5576/supp-2 Table S2 The values of measurement of general plant traits of F. amethystina Each individual was described by four traits: the length of the leaf (cm) –LLe, the length of the stalk (cm) –LS, the length of the panicle (cm) –LPa, the number of spikelets –NS.

## Additional Information and Declarations

### Field Study Permissions

The following information was supplied relating to field study approvals (i.e., approving body and any reference numbers): Collecting the material was approved by the Regional Directors for the Environmental Protection: WPN.6205.170.2014.KLD; WPN-II.6400.14.2014.KW2; WPN.I.6400.21.2014.AC.

### Data Availability

The following information was supplied regarding data availability: The raw data are provided as a Supplemental File.
